# Out of pocket expenditures of patients with a chronic condition consulting a primary care provider in Tajikistan: a cross-sectional household survey

**DOI:** 10.1186/s12913-020-05392-2

**Published:** 2020-06-16

**Authors:** Fabienne B. Fischer, Zulfira Mengliboeva, Gulzira Karimova, Nasrullo Abdujabarov, Helen Prytherch, Kaspar Wyss

**Affiliations:** 1 Swiss Tropical and Public Health Institute, Basel, Switzerland; 2 University of Basel, Basel, Switzerland; 3 Swiss Agency for Development and Cooperation’s Enhancing Primary Health Care Services Project (Project Sino), Dushanbe, Tajikistan

**Keywords:** Out of pocket expenditure (OOP), Primary health care (PHC), Family medicine, Chronic conditions, Chronic disease, Non-communicable disease (NCD), Tajikistan, Central Asia

## Abstract

**Background:**

Within its reform efforts, the Government of Tajikistan is embracing the essential role of primary health care (PHC) in decreasing out of pocket (OOP) expenditures and increasing equity in access to health services. In the light of the increasing burden of disease relating to chronic conditions, we investigated OOP expenditures of patients with chronic conditions within a PHC setting; and if and how those expenditures are impacted by several interventions currently being implemented within Tajikistan.

**Methods:**

A cross-sectional survey among 1600 adult patients who had visited a PHC facility was conducted. The data obtained through interviews were descriptively analysed, and logistic regressions and gamma generalized linear models were performed.

**Results:**

The total OOP expenditures related to a patient’s last visit to the PHC facility were 17.2 USD for those with chronic conditions and 13.9 USD for those visiting due to an acute condition. Adjustment for potential confounders reduced the discrepancy from 3.3 USD to 0.5 USD. This convergence of costs was only observed in districts covered by the Basic Benefit Package (BBP), a governmental pilot project, aiming to standardise exemptions for payment and formal co-payments for health care services. Hence, we found the BBP to have a protective impact for patients with chronic conditions. However, considering the demographics of these patients (older in age, with greater dependency on pensions and social aid, and lower socio-economic status) in combination with the 40% higher utilisation rate of PHC and the high rate of onward referrals to specialists; it is clear that patients with chronic conditions continue to face substantial long-term costs and disadvantages.

**Conclusions:**

After accounting for confounders, patients with chronic and acute conditions faced similar costs related to a single visit to a PHC facility in districts covered by the BBP. However, greater efforts are required to ensure that citizens are well informed about their rights to health care, the BBP and the services that should be provided at no cost at the point of delivery. Moreover, the needs of patients with chronic conditions warrant a more integrative approach that takes long-term expenditures and services beyond the level of PHC into account.

## Background

In its 2014 Global Status Report on Non-communicable Diseases (NCD) the WHO identified NCDs as one of the major health challenges of the twenty-first century [1]. As the leading cause of death globally, NCDs were responsible for 68% of the world’s 56 million deaths in 2012. More than 40% of them were premature deaths under age 70 years and almost three quarters of all NCD deaths occur in low- and middle-income countries [1]. In line with these findings, Tajikistan is also experiencing a shift in disease patterns. While infectious diseases are still common, the prevalence of chronic conditions has been rising steadily since the mid-1990s [2]. In 2016, 69% of all deaths were estimated to be caused by chronic conditions, in particular cardiovascular disease (CVD) (42%), followed by cancer (10%), respiratory diseases (4%), diabetes (2%) and other NCDs (12%) [3].

One of the objectives of the Global NCD Action Plan, highlights the need to address prevention and control for NCDs through primary health care (PHC) and universal health coverage [4]. Accessibility and affordability of health care are greatly dependent upon country level choices regarding the financing modalities. While many high-income countries have a mixture of private and public funding, low- and lower-middle income countries such as Tajikistan still rely heavily on direct financing by households such as out of pocket (OOP) payments by patients to the health care provider [5]. In 2014, 61.7% of the total health expenditure (THE) was found to be comprised of OOP payments [2]. Given that the monthly average wage was estimated to be 115.7 USD in 2016, it is apparent that OOP payments can severely hinder access to health care especially for those of poor socio-economic status (SES) [6]. Patients with chronic conditions can be especially exposed and vulnerable to such costs, as they typically need prolonged care and rely on multiple and regular use of services.

NCD multi-morbidity has been associated with increased OOP expenditures in middle income countries, mainly due to higher expenditures on medicine which can lead to non-adherence to treatment [7, 8]. Even in contexts with social protection schemes in place, household SES influences access to care among patients with chronic conditions, whereby poorer population segments typically face major barriers in obtaining essential services and financial hardship [9]. A study in India in 2012 has provided evidence that OOP payments for chronic conditions are a major driver of impoverishment [10]. While the wider association of poverty and chronic conditions has been well demonstrated, including how the compromised well-being of a chronically ill person might not allow them hold a regular job thus further depriving them of income while they often face higher costs for health care over an extended period of time [11, 12]. Moreover, there is evidence that the poorer segments of society have a generally higher exposure to potential risk factors for chronic conditions [1].

The Government of Tajikistan recognizes the importance of improving access to care and reducing inequities through a more decentralised model of PHC compared to the centralised system focusing on secondary and tertiary hospital care inherited from the Soviet period.

One of the reforms the government initiated was the introduction over the last decade of the basic benefit package (BBP [2]). This package entitles the entire population to free (at point of care) essential PHC and emergency services (e.g. diagnostic consultations, ambulance service) and free secondary and tertiary services for pre-defined “vulnerable” groups based either on social status (e.g. children under one, veterans) or health indication (e.g. patients with tuberculosis). The rest of the population pays formal co-payments (e.g. curative manipulations/services, such as physiotherapy, stitching of wounds etc. are only free for “vulnerable” patients) [2]. The BBP is in the pilot phase and currently being implemented in around a quarter of the districts of the country. However, due to regulatory constraints, the implementation of the BBP package is not without its challenges – most notably the guidelines for exemptions can be confusing for both patient and health care provider resulting in inconsistent application [13].

Since 2003, the “Enhancing Primary Health Care Services” (EPHC) project (previously Project Sino) has been running in Tajikistan, supporting the Government in their efforts to strengthen PHC services by providing training for medical professionals, supporting infrastructures for PHC clinics and strengthening community participation and empowerment.

In the framework of the EPHC project, progress in strengthening PHC services and monitoring of the development of health expenditures have been tracked through a series of household surveys since 2005 [14]. Focusing on only the oldest and the most recent surveys (2005, 2011 and 2014); they have indicated a comparatively high level of satisfaction with the received health care [14–16]. In 2014, informal payments have been reported by 34% of the patients [17]. It has been shown that expenditure for medication is the biggest financial burden for patients. SES-related inequalities have been observed across both households and districts. However, until now there has been no assessment of the differences amongst illness or reasons for consultation.

In the light of the epidemiological transition and the considerable efforts to reform the health system, it is warranted to examine the economic burden of patients with chronic conditions more closely. We anticipated that the utilisation rate of PHC services is increased for patient with chronic conditions, however the intransparent and erratic costing of PHC services make it difficult to hypothesise the effect of the health care reforms on patients with chronic conditions. Further evidence was therefore needed to examine how the strengthening of PHC might have affected the economic burden of patients with chronic conditions.

Consequently, this new iteration of the household study aimed to assess the development of the OOP expenditures and to uncover the situation of patients with chronic conditions, compared with patients visiting the PHC facility for acute conditions.

## Methods

### Study design

To respond to the study objective, we conducted a cross-sectional study among patients that visited PHC facilities between February and May 2016. Following a visit to a PHC centre in the previous 3 months, patients were interviewed at home using a structured questionnaire to find out about their last visit to the health centre, the related expenditure, as well as medicine prescription and usage.

### Study population and sampling methods

The study covered eight districts of rural or semi-rural Tajikistan. In the Khatlon region, Dangara, Hamadoni and Vose districts, as well as Varzob, Faizabad, Rudaki, Shakrinav, Tursunzade as part of the Region of Republican Subordination (RRS). Six of the eight study districts are included in the BBP pilot project, except Rudaki and Vose. The study population were adult users of PHC services (health centres or rural health centres). A multi-level sampling strategy was applied to select the facilities and the number of respondents in each location. The number of PHC facilities to be included in the study for each district was chosen proportionally to the total number of PHC facilities in the respective district. Additionally the PCH facilities were selected to represent both accessible and remote facilities. All facilities were staffed with at least one family doctor (FD). In a second step, the total number of respondents per PHC facility was chosen proportionally to the total number of patients’ visits in the previous year to this facility. The respondents were then selected using a systematic random sampling technique from the registries at the health centre.

The sample size of 1600 patients and the sampling methodology was aligned to the previous studies conducted in 2011 and 2014 to enable meaningful comparison and establishment of a time-trend [15, 16]. The sample size was based on the requirement to detect at least 10% difference in total OOP expenditure and a 7% difference in the access to medicine across SES status using a power of 90% and a significance level of 5% with possible stratification along the eight districts.

### Data collection and analysis

Selected patients were visited at home and interviewed after obtaining oral consent. The interviewers were locals with previous experience in conducting surveys and received additional training for this study. The data were collected using a questionnaire adapted from the previous studies with small updates to facilitate analysis on patients with chronic conditions (see Additional file 1 for the complete questionnaire) [16]. In addition to demographic variables, the questionnaire aimed to assess the OOP expenditure, experiences with the FD and prescribed and bought medicine. The inquiry about OOP expenditure was divided into six categories: i) formal fees for consultation; ii) informal payments to the FD or family nurse; iii) the value of non-monetary gifts given; iv) the amount of money spent to reach the health centre; v) the amount of money spent for travel to obtain medicine; vi) the expenditures for the prescribed medicine. Furthermore, the participants’ SES was estimated based on the assessment of variables including income source, housing infrastructure, meat consumption and similar factors using principal component analysis to compute a household asset index.

Data were collected in May 2016 with tablets and the software package CSPro. Independent monitoring activities were pursued to ensure data quality and adherence to the described methodology.

The data were analysed using Stata/IC 14.0 software. For univariate analysis, associations were tested for statistical significance using the chi-squared test for binary and categorical variables. This test was also used to assess linear associations (dose-response relationships). For continuous variables, the Wilcoxon rank-sum test as well as the Kruskal-Wallis test were used for testing between two or more groups respectively. For binary variables logistic regressions were run and ordinal logistic regressions for ordinal variables. To assess the different amounts of expenditure (per category and overall) and their determinants, generalized linear models (GLM) for Gamma-distributed variables were used. The Gamma-distribution can be used to model monetary variables with their typical right-skewed distribution, and GLM-models for Gamma-distributed variables have been shown to be useful for the prediction of average costs and have been applied before in health care settings [18, 19]. Regression models were run according to a stepwise approach. In a first step, an empty model was run only including consultation reason as explanatory variable. In a second step, the model was adjusted for sex and age. Finally, a full model was developed with included reason for consultation (acute, chronic), age categories, sex, coverage status by BBP, region (Khatlon, RRS), SES status and number of doctors’ visits in the last 12 months. After identifying the strong confounding effects of the BBP project on our outcome variable, we have decided to further stratify the full model (excluding coverage status by BBP) by BBP status. Stratified findings are only reported where significant differences were observed to improve the readability of the findings.

The OOP spending was always assessed two-fold: (i) The percentage of patients reporting to have spent money (ii) The mean of expenditure of all patients who have spent money. The amount of money is presented in USD, the currency exchange rate of 7.87 Tajik somoni (TJS) per one US dollar (USD) as of May 2016 was used.

In the questionnaire patients were divided by their reason for consultation, differentiating between “acute disease”, “chronic disease”, “pregnancy”, “injury or poisoning” with the option of “other”. For the analysis patients not visiting for either acute or chronic conditions were omitted as they were difficult to compare: many of them were pregnant women who represent a different subpopulation, while the population visiting for injury and poisoning was rather small.

To complement the information collected through the household survey, 18 in-depth interviews were conducted. The interviews were transcribed and translated into English and thematically coded and analysed using the software package MAXQDA 12. The information from these interviews is drawn on where it is helpful for interpreting the collected data and results, but is not otherwise discussed in detail.

## Results

### Characteristics of the total sample population

Among all patients contacted, 487 (30% of the anticipated sample size) did not agree to be interviewed at home and were replaced by other study participants.

Through the weighted sampling method the following numbers of patients were interviewed in the districts Dangara 12% (*N* = 191); Varzob 10% (*N* = 166); Tursunzade 27% (*N* = 439); Shahrinav 5% (*N* = 79); Vose 16% (*N* = 250); Hamadoni 11% (*N* = 183); Rudaki 15% (*N* = 235) and Faizabad 4% (*N* = 57).

The majority of the interviewed patients were female (85%). This gender difference persisted even when accounting for pregnancy-related visits (76 to 24%). The median age was 31 years old with an average of 11 years of education. The main reason for consultation was pregnancy with 38% (*N* = 609), followed by chronic conditions with 34% (*N* = 542) and acute diseases (23%, *N* = 363) (Table 1). Another reason for consulting the FD was the need for a medical certificate, e.g. for meeting the legal requirements for a wedding.

**Table 1:** Reason for consultation at the last PHC centre visit, Tajikistan, 2016

Reason for consultation	N	
Acute conditions	361	(23%)
Chronic conditions	539	(34%)
Pregnancy	612	(38%)
Injury/Poisoning	41	(3%)
Others	47	(3%)
Total	1600	(100%)

Patients with chronic conditions were found to have a higher age (median 50 years old compared to 35 years), higher dependency on pensions and social aid (21% compared to 13%) and of lower SES (Table 2). There was no difference in the distribution of patients with chronic conditions by status of BBP coverage. Differences were found in terms of geography: of all patients living in the Khatlon region, 70% reported having a chronic condition, while in the RRS the prevalence was only 51%.

**Table 2:** Demographics of patients consulting the doctor for acute and chronic conditions, Tajikistan, 2016

Variables		Acute		Chronic		Total	
Number of respondents		361	(40%)	539	(60%)	900	(100%)
Gender	Female	270	(75%)	414	(77%)	684	(76%)
	Male	91	(25%)	125	(23%)	216	(24%)
Median age		35		50		45	
Median number of years of education		9–11		9–11		9–11	
BBP	not covered	99	(27%)	171	(32%)	270	(30%)
	covered	262	(73%)	368	(68%)	630	(70%)
Region	Khatlon	125	(35%)	291	(54%)	416	(46%)
	RRS	236	(65%)	248	(46%)	484	(54%)
SES	0–20% Poorest	81	(22%)	147	(27%)	228	(25%)
	20–40% Poor	71	(20%)	133	(25%)	204	(23%)
	40–60% Middle	69	(19%)	102	(19%)	171	(19%)
	60–80% Wealthy	68	(19%)	91	(17%)	159	(18%)
	80–100% Most wealthy	72	(20%)	64	(12%)	136	(15%)
Median number of visits to a FD in the past 12 months		3		5		4	

Patients with chronic conditions consulted the FD more often than the other patient groups with a median of 5 visits in the last 12 months compared to 3, for patients with acute conditions. Furthermore, 60% of the patients with chronic conditions stated that their last visit was a follow-up visit to previous consultations. It should be noted that only the costs related to the most recent consultation were explored.

**Fig. 1:**
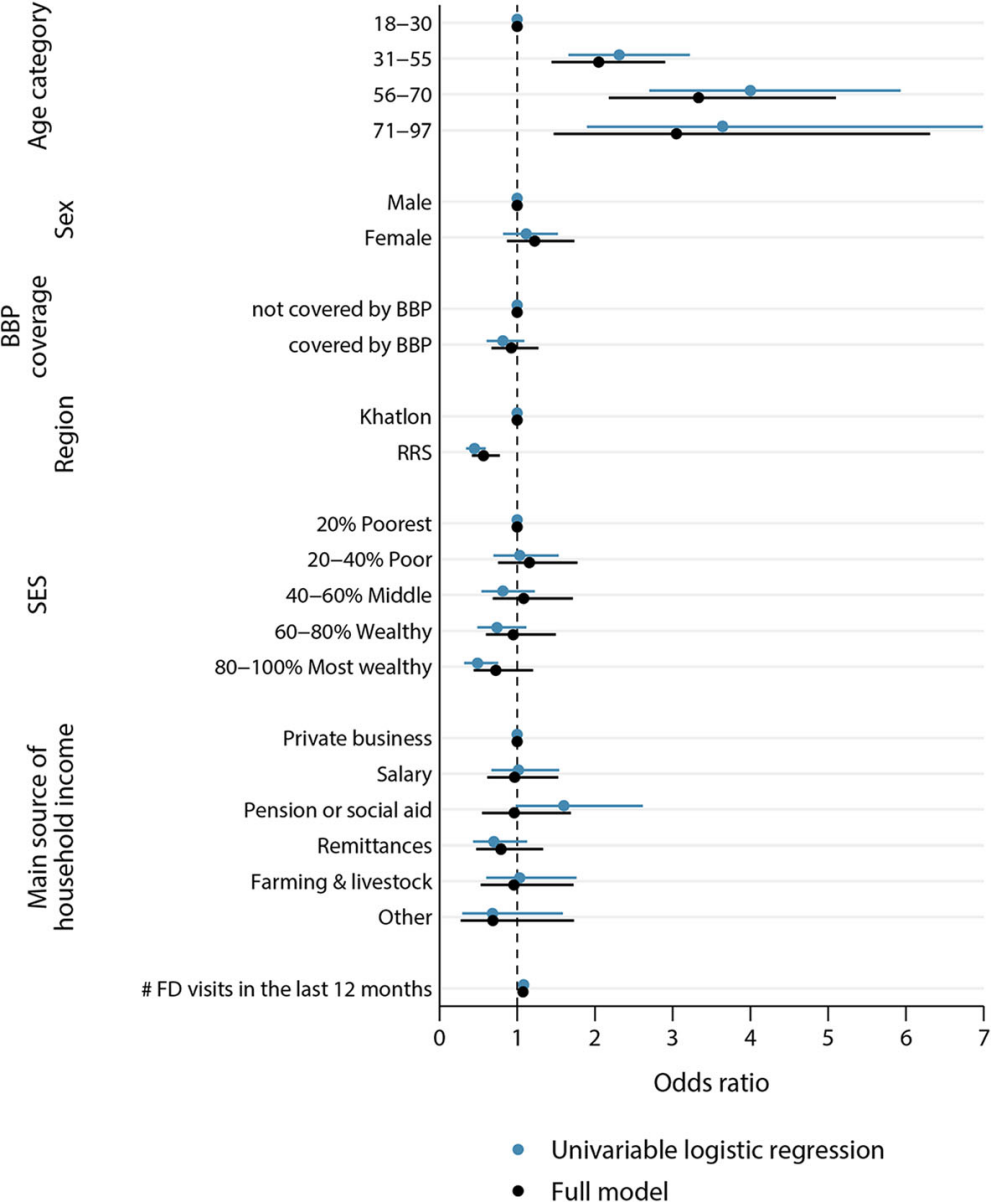
Logistic regression on consultation reason at the primary health care centre (0 = acute conditions, 1 = chronic condition), univariable and multivariable model, Tajikistan, 2016

After adjustment for other explanatory factors, only age and region were shown to be significant predictors for chronic conditions, as shown in Fig. 1.

Overall respondents with chronic conditions rated their own health as worse than patients with acute conditions, with 28% rating their health as poor or very poor compared to 13% of patients with acute conditions.

### Access to PHC services and perceived quality of care

Of the 900 patients visiting the FD for either chronic or acute conditions, 74% perceived the distance to the PHC facility as acceptable and easy to access. The satisfaction with the distance did not correlate with the time needed to reach the PHC facility. Of the 90 patients, which reported that the PHC facility was too far and difficult to access 72% were suffering from chronic conditions. However, age was found to be a confounder of this association, as mostly patients older than 56 years expressed difficulties accessing the PHC facility (56–70 years old: aOR 0.4, *p* < 0.01; 71+ years old: aOR 0.3, *p=* 0.02).

Patients with chronic conditions were more frequently referred to a specialist (49%) than those suffering from an acute problem (41%). Even after accounting for other factors, being chronically ill or aged between 21 and 55 years increased the odds of being referred. However, the largest predictor was living in RRS region where the adjusted odds ratio (aOR) to referral was 3.5.

The overall satisfaction with the care received was reported to be high. The majority of patients with chronic disease were either very satisfied (41%) or satisfied (56%), similar to the patients with acute conditions (47 and 50%).

### Out of pocket expenditures

#### Percentage of paying patients

Patients with chronic conditions more often reported no expenditures at all (16%) compared to patients with acute conditions (10%). This difference could also be seen in all regression models (unadjusted, partially adjusted and full model, see Table 3). However, stratification for BBP coverage showed that the beneficial effect for patients with chronic conditions only held true in districts covered by the BBP (aOR 0.5, *p* < 0.01).

**Table 3:** Odds ratio of frequency of paying (0 = no money paid, 1 = money paid), for patients with chronic condition as consultation reason and its associated *p*-value from different gamma GLM models, Tajikistan, 2016

	Unadjusted		Adjusted for age and sex		Full model	
	OR	***p***-value	aOR	***p***-value	aOR	***p***-value
**Formal**	1	0.84	1.2	0.31	1.3	0.26
**Informal**	1.1	0.52	1.3	0.33	1.4	0.14
**Medicine**	0.9	0.69	0.9	0.40	0.8	0.17
**Travel to a FD**	0.7	0.02	0.7	< 0.01	0.7	0.07
**Travel to obtain medicine**	1	0.88	0.9	0.41	0.9	0.43
**Total OOP**	0.6	0.01	0.5	< 0.01	0.6	< 0.01

Among all patients with either chronic or acute conditions, 18% reported paying formal fees for consultation. Thirteen percent of patients with chronic conditions and 12% of patients with acute conditions reported informal payment. Only a small number of patients reported giving non-monetary gifts (1%). Seventy-one percent and 73% of chronic and acute conditions respectively had to procure medicine in relation to their last consultation at the PHC facility. Forty-five percent of each patient group reported additional expenditure for travel to obtain the medicine. Travel expenses varied slightly across conditions; 24% of patients with chronic conditions reported incurring expenses for travel to the PHC facility, while 32% of patients with acute conditions reported such expenses. In the model including other explanatory variables, regional differences accounted for some of this discrepancy - as patients from the RRS region had higher odds of paying for transport to the PHC facility (Table 3). No differences between the patient groups were observed for incurring other types of expenditures.

#### Amount of money spent

Patients with chronic conditions reported an overall OOP expenditure of 17.2 USD related to their visit to the FD. OOP expenditures of patients with acute conditions were lower at 13.9 USD. However, this observed difference was not statistically significant and taking other explanatory variables into account diminished it further, resulting in 16 USD total OOP expenditures for chronic conditions and 15.5 USD for acute conditions respectively (see Table 4). Stratification by BBP coverage, showed that patients with chronic conditions pay significantly more than patient with acute conditions in districts not covered by the BBP (27.3 USD and 17.7 USD, *p* = 0.04) even after adjustment for the other variables.

**Table 4:** Amount of money paid, if payment has been reported: Coefficients of chronic condition as consultation reason and its associated *p*-value from different logistic regression models, Tajikistan, 2016

	Unadjusted			Adjusted for age and sex			Full model		
	Acute*	Chronic*	***p***-value	Acute*	Chronic*	***p***-value	Acute*	Chronic*	***p***-value
**Formal**	4.1	3.8	0.90	7	3.2	0.02	4.8	3.2	0.23
**Informal**	1	1.2	0.52	1.1	1.2	0.59	1.1	1.2	0.88
**Medicine**	16.1	20.8	0.07	16.6	20.5	0.16	21	23.4	0.42
**Travel to a FD**	0.8	0.9	0.51	0.8	0.9	0.63	0.9	0.9	0.90
**Travel to obtain medicine**	2.4	2.8	0.32	2.3	2.8	0.30	2.4	2.8	0.27
**Total OOP**	13.9	17.2	0.12	14.5	16.7	0.31	15.5	16	0.82

*Values in USD

Patients with chronic and acute conditions reported similar amounts of informal OOP expenditure for consultation (1.2 USD and 1.1 USD), as well as similar expenditures for the travel to obtain medicine (2.8 USD and 2.4 USD). The value of non-monetary gifts was not evaluated due to the negligible percentage of patients reporting this expenditure.

Acute and chronically ill patients paid a similar amount for consultations (4.1 USD and 3.8 USD). Even after adjusting for all variables, only the age group of 56 to 70 years old and living in the RRS region showed increased odds for higher amount of formal expenditures, while there were no differences between patients with acute and chronic conditions (Table 4).

Patients with chronic conditions that were not covered by the BBP had significantly higher expenses for medicine (45.4 USD) than patients with acute conditions 29.6 USD, *p* = 0.05), while there was no significant difference in districts covered by the BBP. Medicine costs greatly exceed the expenditures for all other services.

### Medicine prescription and purchase

The percentage of patients leaving the FD with a prescription for at least one drug was similar for those suffering from a chronic condition (88%) and those with an acute condition (85%). However, the fully adjusted regression model showed decreased odds for medicine prescription for patients with chronic conditions (Table 5). After stratification by BBP coverage, only chronically ill patients living in districts covered by the BBP had significantly decreased odds for medicine prescription (aOR 0.5, *p* = 0.02).

**Table 5:** Odds ratios for medicine prescription and purchase for patients with chronic condition as consultation reason and its associated *p*-value from different gamma GLM models, Tajikistan, 2016

		Unadjusted		Adjusted for age and sex		Full model	
		OR	***p***-value	aOR	***p***-value	aOR	***p***-value
**Has medicine been prescribed?**		0.7	0.12	0.68	0.07	0.61	0.03
**Type of drug prescribed:**	**IV**	1.9	< 0.01	1.6	< 0.01	1.4	0.05
	**Non-IV**	1.2	0.34	1.1	0.55	1.1	0.65
	**Antibiotics**	1	0.91	1.1	0.65	1	0.86
	**Vitamins**	10.2	0.88	1.1	0.63	1.1	0.71
**Received ≥ 5 prescriptions**		1.5	< 0.01	1.55	0.01	1.5	0.01
**Has all medicine been obtained?**		1.3	0.30	1.5	0.06	1.5	0.14
**Non-prescription medicine**		1.5	< 0.01	1.45	0.07	1.3	0.15
**Supplementary treatment**		1.8	< 0.01	1.6	< 0.01	1.3	0.18

Overall a high prescription rate for intravenous (IV) injections, such as “drips” (39% for acute conditions and 55% for chronic), other types of injections ([intramuscular, subcutaneous or intradermal injections or others], 51 and 54%), antibiotics (both 50%) and vitamins (both 60%) was observed. Patients with chronic conditions reported a significantly higher prescription rate for IV-injections; this difference persisted in the regression model even when all explanatory variables were taken into account (Table 5).

Of all the 774 patients being prescribed medicine, 9% of those with chronic conditions and 7% of patients with acute conditions reported not to have obtained any or only part of the drugs. This was mainly due to a lack of money – cited by 78% of patients with chronic conditions and 63% of patients with acute conditions. In districts covered by the BBP patients with chronic conditions were more likely to not obtain any or all prescribed medicine (aOR 2.2, *p* = 0.03).

Patients with chronic conditions seemed to rely more on drugs that were not prescribed by the FD (33%) and supplementary treatment (28%) than patients with acute conditions (25% reported using non-prescribed drugs and 18% supplementary treatment). However, both these effects diminished after taking other explanatory variables into account. Non-prescribed medicine usage was highest for females and the age group between 56 and 70 years, while the use of supplementary treatment was more widespread in older age groups and in Khatlon region. Supplementary treatment included the use of herbs (32%), traditional treatment (28%), praying (19%), as well as consultations with other doctors (17%).

## Discussion

### Access to PHC services and quality of care

The satisfaction expressed with PHC services was high. These findings are in line with the results from the previous studies [15, 16]. It is difficult to judge whether this is a true perception of the care received or if the high rating could be attributed to the general unwillingness to criticise for fear of possible consequences. Other studies have suggested a potential gratitude bias to limit the reporting of negative opinions [20].

### OOP expenditures for one visit to the FD

To effectively analyse the financial burden on households relating to a PHC consultation, the expenditures have to be viewed in context of the financial capacities of the Tajik population. The monthly average wage of those in paid employment in Tajikistan in May 2016 was estimated to be 115.7 USD [6]. However, there are strong discrepancies between rural and urban areas, with households in rural areas and especially those relying on agriculture or the informal economy being typically much poorer. As this study has been conducted in mostly rural settings, it has to be assumed that the household income among the study population is substantially lower than the estimated country average. Further, since 2015 the international poverty line was set at 1.9 USD (PPP) a day, equalizing to 57.8 USD a month. In was estimated that in 2015 31.3% of the population in Tajikistan lived below the national poverty line [21]. In this context, the findings of the study show that OOP expenditures for health remain a substantial burden to households in rural Tajikistan with one visit to the PHC facility amounting to one seventh of the average monthly wage or almost a quarter of the income of the patients below the poverty line.

Consistent with the findings of the survey in 2014 focusing on medical expenditures and studies from the neighbouring Kyrgyzstan and other lower income countries, medicine costs still account for the biggest proportion of OOP expenditures relating to PHC use [16, 22, 23]. Most likely, this finding is attributable to insufficient supplies of pharmaceuticals from the essential drug list and poor prescription patterns, such as over-prescription of expensive injections, prescription of antibiotics for non-bacterial causes and over-prescription in general [2]. The high number of prescribed antibiotics and injections follows a similar pattern to other countries in Central Asia, China and beyond [24]. In the in-depth interviews, patients reported that they seem to put more trust in the effectiveness of injections than pills and request them from providers. This observation would be in line with studies from other developing countries such as Uganda, Indonesia and Pakistan, who have reported that injections are perceived as faster, more efficient or even safer than pills [25, 26]. It has also been shown that the informal payments in particular seem to be tied to small services, e.g. one injection for one TJS, so financial interest to prescribe injections could be in play on the provider side.

Informal payments remain a common practice in Tajikistan with one in eight patients reporting to have paid on an informal basis. However, it seemed difficult for patients to talk about those spendings, but also to differentiate them from the formal payments for consultation. Therefore, the actual percentage of patients reporting to have paid on an informal basis might be underestimated. Based on the in-depth interviews, we suggest that health care providers rarely request informal payment, but patients seem to provide it on their own accord. The voluntary nature of these informal payments is arguably questionable. Reasons for payment could be divided into four categories: i) payment out of gratitude for good health to ensure emotional and spiritual well-being; ii) attempt to ensure continuous and good quality of care; iii) perception that the time and material used by the provider must be paid; iv) abiding to traditions on informal payments. Informal payments and expectations are rarely discussed with the FD.

The fact that patients from all districts reported payment on an informal basis (even though the BBP aims to formalise all payments) and patients from districts not covered by the BBP reported a higher rate of formal payments, even though in these districts access to all health services should be free [2] suggest that patients seem to lack awareness of their right to free PHC and therefore cannot question the appropriateness of expenditures they face. This is supported by the in-depth interviews conducted subsequently to the data collection. Consequently and as to reduce the financial burden to households, it appears important that the population and health service users are adequately informed on their entitlements.

### Experiences of patients with chronic conditions at PHC level

As only a single visit to the PHC centre was assessed, large differences in OOP expenditures for acute and chronic patients were not expected. The greater financial burden for patients with chronic conditions stem from i) higher utilisation rate of PHC services; ii) the need for diagnostics tests, medicines and treatments, beyond the capacities of PHC in Tajikistan.

Indeed patients with chronic and acute conditions reported similar OOP expenditures related to a single visit PHC facility, if controlled for confounding. Most discrepancies between patients visiting for acute and chronic conditions diminish, when other variables such as age or gender are taken into account. Conversely, patients with chronic conditions were more likely to not report any expenditure at all than patients with acute conditions.

However, further analysis showed that these effects are strongly confounded by the BBP and only hold true in districts covered by the BBP.

#### The impact of the basic benefit package

The fact that the BBP exempts certain vulnerable groups including chronic conditions, such as diabetes mellitus, HIV/AIDS or tuberculosis, from payment suggest a better protection from costs in districts covered by the BBP. Indeed, patients with chronic conditions reported paying significantly less often and also paying a smaller amount compared to patients with acute conditions, if covered by the BBP package. Additionally patients pay a similar amount for medicine regardless of consultation reason if covered by the BBP. This suggests that the essential drugs which can be obtained at the PHC level to treat chronic conditions, are not disproportionately expensive [27]. However, it should be noted that the costs of drugs, prescribed and obtained at secondary or tertiary level, which are likely more expensive, have not been assessed in this study. Conversely, patients with chronic conditions reported significantly higher expenditures if not covered by the BBP. The lowering of the medicine costs and total expenditures suggests a protective impact of the BBP for patients with chronic conditions. However, patients with chronic conditions in BBP covered districts were also more likely to forego at least some of the prescribed medicine than patients not covered by the BBP, and reported a lack of money as the reason. This finding is counterintuitive, however we see three possible explanations why the BBP may not have a protective effect: i) inconsistent implementation due to the complicated lists of free or partially covered services; ii) the needs of patients with chronic conditions for drugs which are not on the essential medicine lists and less likely to be covered by the BBP; iii) the fact that the non-availability of drugs can happen regardless of the payment modality [13].

#### Medicine prescription and purchase

In terms of overall medicine purchase, a relatively high percentage of patients with chronic conditions reported obtaining all the drugs that were prescribed. However, based on this information, it cannot be concluded that procuring medicine is not problematic. Several patients reported being unable to forego their medicine, as it was essential to stop their health from completely deteriorating. Most of the interviewed patients gave their health very high priority above all other necessities. Generally, the patients were ready to take all necessary measures to obtain the medicine: borrowing money from family members, accumulating debts, impoverishment, skimping on other needs [28]. Lastly, the percentage of patients buying the medicine does not reflect the medicine usage. Patients reported rationing the drugs and not strictly adhering to the prescription recommendation. Similar behaviours in obtaining and saving funds for health care have been described in Mongolia [29].

#### Higher PHC service utilisation rate

Even if the BBP package seemed to alleviate some of the financial burden of patients with chronic conditions, our findings do not allow the conclusion that patients with chronic conditions do not suffer substantial disadvantages when seeking appropriate health care. This study only reflects the costs of one visit to the doctor, while chronic conditions often require long-term treatment. Hence, this study can only estimate the true recurring health care costs. Because patients with chronic conditions reported visiting the doctor more often (on average 5 times in the past 12 months compared to 3), the annual spending for PHC is expected to be 40% higher for patients with chronic conditions. This assumption would be in line with other studies: patients with NCDs are associated with an increased utilisation of health services [7] and a study in India has shown a high correlation of yearly OOP expenditures and chronic illnesses [30]. The prolonged treatment and management of chronic conditions further puts those patients under stress.

The lack of differences between expenditures regarding a single visit to the FD can also be attributed to the fact that PHC in Tajikistan only provides basic services and medicine [2]. The essential list of drugs to treat chronic diseases at PHC level has specifically been designed to be affordable [27]. However, many diagnostic tests or extended treatments have to be done upon referral and are handled by a specialist and/or at hospital level. Patients with chronic conditions are likely to use and benefit from the same resources as patients with acute conditions and seek additional treatment outside of the PHC setting, which is also reflected in the higher referral rate, as found in this study. The costs occurring from these referrals (including diagnostic procedures) could not be assessed by means of this study.

#### Confounding effects: the demographics of patients with chronic conditions

Based on the overall OOP expenditure patients with chronic conditions pay more in districts not covered by the BBP, while the BBP seems to level the OOP expenditures. However, inequity in accessing health care is not yet achieved, as the average patient consulting the FD for chronic conditions differs from one consulting for an acute condition: Patients in this study with chronic disease were of higher age, more likely to be dependent on social support or pensions, and more likely to belong to a lower SES group. The concurrence of these characteristic suggests that patients with chronic conditions were more likely to lack resources to pay for their needs or need to spend higher proportions of their monthly income on health care.

This concurrence of disease status, age and SES status is problematic, as these population groups are already vulnerable to costs on an individual level. A study from 2013 has shown that reduction of poverty among the elderly is more dependent on private transfers (i.e. remittances, borrowed money from family) rather than public transfers (i.e. social or old age pension), highlighting the importance of a social network and potentially insufficiency of the public protection system [31, 32].

Various studies indicate inequities of OOP expenditures with substantially higher relative expenditures among low-income households [10, 14, 33–35]. This study supports those findings. While there was no association between absolute OOP expenses and SES, the relative share of OOP expenditures is higher for low-income or low-SES households. A study in 2010 reported an association of the ability to pay with a higher amount of OOP expenditures related to hospitalization [36]. Another study from 2015 in Malawi showed the highest OOP expenditures for the wealthiest, but the poorer spend the bigger proportion of their total income [37]. It is suggested that a similar pattern is here in place as well: Reported OOP expenditures did not vary significantly among SES status, but patients with chronic conditions (more often of lower SES) rated their own health worse than patients with acute conditions. It has been established that the OOP expenses are a risk for impoverishment of households, which in turn hinder the access to adequate treatment, leading to a vicious cycle of poverty and disease [1, 38, 39]. Due to this correlation of poverty, dependence and chronic conditions, inequality in terms of OOP expenditures for PHC services might not present themselves in the form of higher costs, but rather the patient suffers from the stress and pressure to acquire money to pay the services and the need for referral and additional treatment, to the basic services the of the PHC provide.

### Limitations

Almost one third of patients initially contacted declined participation, which could have led to a selection bias. The non-respondent rate is likely explained by the following: women were not allowed to participate without their husband or mother-in-law present, men do not engage with strangers coming to their house in fear of being drafted into the military, people were afraid of repercussions if they do not give the “correct” reply (anecdotally, we have been told of “fake surveys” being conducted) and talking about money and especially the lack thereof is a very private and sensitive topic. The strong skew in the sampling distribution of female to male participant towards women can mainly be attributed to the high dependency of Tajikistan on remittances, resulting in many men emigrating to other countries, leaving the women behind [40, 41].

It needs also to be noted that the patients that were interviewed were the ones, which could afford to have made at least one visit to the health centre in the previous 3 months. In this study we only investigated the direct costs occurred when visiting a health facility, but could not assess the indirect costs (e.g. absenteeism/productivity losses) of the patients, nor their care givers in the family. Those indirect costs are also likely to heavily affect patients with chronic conditions.

The study is based on interviews and hence, self-reported data. Self-reported data are always subject to recall and reporting bias. To limit recall bias only patients that had visited a health facility in the previous 3 months were included. For a few health facilities, the data collection team could not find enough patients within this timeframe and extended up to 5 months, which might have increased the recall bias.

The reported rates of referral in this study were very high when compared to estimates of previous studies [42]. Possibly the question was misunderstood, and perhaps even the use of a diagnostic test was considered by respondents as a referral to a specialist.

## Conclusions

In conclusion, this study showed that after adjustments, patients with chronic and acute conditions incur similar costs related to a single visit to the FD at PHC level for districts involved in the BBP pilot. The BBP and the strengthening of PHC services seemed to have a positive effect on the expenditures of patients with chronic conditions in regards to one visit to the FD and it will be important to ensure citizens are well informed about its intention and their rights. Still, patients with chronic conditions often belong to a vulnerable subpopulation (the poor and elderly). Moreover, most of the potential financial hardships for patients with chronic conditions are likely to be related to the long-term implications of these conditions: Prolonged treatment, higher utilisation rate of health services and need for services beyond the current possibilities of PHC in Tajikistan. Hence, an integrative approach that accounts for long-term expenditures and services beyond the level of PHC is needed to protect patients with chronic conditions.

## Supplementary information

**Additional file 1.** Questionnaire [16].

## Data Availability

The datasets generated and/or analysed during the current study are available from the corresponding author on reasonable request with permission of the Tajik MoHSP.

## Abbreviations

aOR: Adjusted odds ratio
BBP: Basic Benefit Package
CVD: Cardiovascular disease
FD: Family doctor
Gamma GLM: Gamma generalized linear model
MoHSP: Ministry of Health and Social Protection (Tajikistan)
NCD: Noncommunicable disease
OOP: Out of pocket
OR: Odds ratio
PHC: Primary health care
RRS: Region of Republican Subordination
SDC: Swiss Agency for Development and Cooperation
SES: Socio-economic status
THE: Total health expenditure
TJS: Tajik somoni
USD: US dollar
WHO: World Health Organization

## References

[1] Mendis S (2014). Global status report on noncommunicable diseases 2014.

[2] Khodjamurodov G, Sodiqova D, Akkazieva B, Rechel B (2016). Tajikistan: health system review. Health Syst Transit.

[3] WHO Noncommunicable diseases country profiles 2018. Geneva: World Health Organization; 2018.

[4] World Health Organization (2013). Global action plan for the prevention and control of noncommunicable diseases 2013–2020.

[5] Gottret P, Schieber G. Health financing revisited : a practitioner's guide. Washington, DC: World Bank; 2006. https://openknowledge.worldbank.org/handle/10986/7094. Accessed 5 Dec 2019.

[6] Tajikistan average monthly wages. Trading Economics; 2016. https://tradingeconomics.com/tajikistan/wages. Accessed 21 Nov 2016.

[7] Lee JT, Hamid F, Pati S, Atun R, Millett C (2015). Impact of noncommunicable disease multimorbidity on healthcare utilisation and out-of-pocket expenditures in middle-income countries: cross sectional analysis. PLoS One.

[8] Sum G, Hone T, Atun R, Millett C, Suhrcke M, Mahal A, Koh GC-H, Lee JT (2018). Multimorbidity and out-of-pocket expenditure on medicines: a systematic review. BMJ Glob Health.

[9] Goeppel C, Frenz P, Grabenhenrich L, Keil T, Tinnemann P (2016). Assessment of universal health coverage for adults aged 50 years or older with chronic illness in six middle-income countries. Bull World Health Organ.

[10] Bhojani U, Thriveni B, Devadasan R, Munegowda C, Devadasan N, Kolsteren P, Criel B (2012). Out-of-pocket healthcare payments on chronic conditions impoverish urban poor in Bangalore, India. BMC Public Health.

[11] Jamison D, Gelband H, Horton S, Jha P, Laxminarayan R, Mock C, Nugent R (2017). Economic burden of chronic ill health and injuries for households in low-and middle-income countries. Disease Control Priorities: Improving Health and Reducing Poverty.

[12] Koolhaas W, van der Klink JJ, de Boer MR, Groothoff JW, Brouwer S (2014). Chronic health conditions and work ability in the ageing workforce: the impact of work conditions, psychosocial factors and perceived health. Int Arch Occup Environ Health.

[13] Jacobs E (2019). The politics of the basic benefit package health reforms in Tajikistan. Global Health Res Pol.

[14] Tediosi F, Aye R, Ibodova S, Thompson R, Wyss K (2008). Access to medicines and out of pocket payments for primary care: evidence from family medicine users in rural Tajikistan. BMC Health Serv Res.

[15] Schwarz J, Wyss K, Gulyamova ZM, Sharipov S (2013). Out-of-pocket expenditures for primary health care in Tajikistan: a time-trend analysis. BMC Health Serv Res.

[16] Donadel M, Karimova G, Nabiev R, Wyss K (2016). Drug prescribing patterns at primary health care level and related out-of-pocket expenditures in Tajikistan. BMC Health Serv Res.

[17] Donadel M (2014). Access barriers and determinants of household out-of-pocket expenditures for primary health care in Tajikistan in 2014 and a time trend analysis between 2005 and 2014. MSc thesis*.* Swiss Tropical and Public Health Institute and University of Basel.

[18] Gregori D, Petrinco M, Bo S, Desideri A, Merletti F, Pagano E (2011). Regression models for analyzing costs and their determinants in health care: an introductory review. Int J Qual Health Care.

[19] Moran JL, Solomon PJ, Peisach AR, Martin J (2007). New models for old questions: generalized linear models for cost prediction. J Eval Clin Pract.

[20] Bernhart MH, Wiadnyana IG, Wihardjo H, Pohan I (1999). Patient satisfaction in developing countries. Soc Sci Med.

[21] Global Poverty Working Group: Poverty headcount ratio at national poverty lines (% of population). The World Bank; 2015. https://data.worldbank.org/indicator/SI.POV.NAHC?locations=BG. Accessed 21 Nov 2016.

[22] Akkazieva BJ (2016). Melitta, Temirov a: long-term trends in the financial burden of health care seeking in Kyrgyzstan, 2000–2014.

[23] Cameron A, Ewen M, Ross-Degnan D, Ball D, Laing R (2009). Medicine prices, availability, and affordability in 36 developing and middle-income countries: a secondary analysis. Lancet.

[24] Li Y, Xu J, Wang F, Wang B, Liu L, Hou W, Fan H, Tong Y, Zhang J, Lu Z (2012). Overprescribing in China, driven by financial incentives, results in very high use of antibiotics, injections, and corticosteroids. Health Aff (Millwood).

[25] Janjua NZ, Hutin YJ, Akhtar S, Ahmad K (2006). Population beliefs about the efficacy of injections in Pakistan's Sindh province. Public Health.

[26] Van Staa A, Hardon A (1996). Injection practices in the developing world: results and recommendations from field studies in Uganda and Indonesia.

[27] World Health Organization (2010). Package of essential noncommunicable (PEN) disease interventions for primary health care in low-resource settings.

[28] Hutchison C, Khan MS, Yoong J, Lin X, Coker RJ (2017). Financial barriers and coping strategies: a qualitative study of accessing multidrug-resistant tuberculosis and tuberculosis care in Yunnan, China. BMC Public Health.

[29] Janes CR, Chuluundorj O, Hilliard CE, Rak K, Janchiv K (2006). Poor medicine for poor people? Assessing the impact of neoliberal reform on health care equity in a post-socialist context. Glob Public Health.

[30] Brinda EM, Kowal P, Attermann J, Enemark U (2015). Health service use, out-of-pocket payments and catastrophic health expenditure among older people in India: the WHO study on global AGEing and adult health (SAGE). J Epidemiol Community Health.

[31] Falkingham J, Vlachantoni A (2012). Social protection for older people in Central Asia and the South Caucasus. Social Protection for Older Persons: Social Pensions in Asia.

[32] Tolla MT, Verguet S, Bekele A, Amenu K, Abdisa SG, Johansson KA, Norheim OF (2017). Out-of-pocket expenditures for prevention and treatment of cardiovascular disease in general and specialised cardiac hospitals in Addis Ababa, Ethiopia: a cross-sectional cohort study. BMJ Glob Health.

[33] Chuma J, Maina T (2012). Catastrophic health care spending and impoverishment in Kenya. BMC Health Serv Res.

[34] Rahman MM, Gilmour S, Saito E, Sultana P, Shibuya K (2013). Health-related financial catastrophe, inequality and chronic illness in Bangladesh. PLoS One.

[35] Ruger JP, Kim HJ (2007). Out-of-pocket healthcare spending by the poor and chronically ill in the Republic of Korea. Am J Public Health.

[36] Habibov N (2010). Hospitalization in Tajikistan: determinants of admission, length of stay, and out-of-pocket expenditures. Results of a national survey. Int J Health Plann Manag.

[37] Wang Q, Fu AZ, Brenner S, Kalmus O, Banda HT, De Allegri M (2015). Out-of-pocket expenditure on chronic non-communicable diseases in sub-Saharan Africa: the case of rural Malawi. PLoS One.

[38] Khan JA, Trujillo AJ, Ahmed S, Siddiquee AT, Alam N, Mirelman AJ, Koehlmoos TP, Niessen LW, Peters DH (2015). Distribution of chronic disease mortality and deterioration in household socioeconomic status in rural Bangladesh: an analysis over a 24-year period. Int J Epidemiol.

[39] Alwan A (2011). Global status report on noncommunicable diseases 2010.

[40] Glenn R (2009). Abandoned wives of Tajik labor migrants: IOM study on the socio-economic characteristics of abandoned wives of Tajik labor migrants and their survival capabilities. International Organization for Migration.

[41] World Bank staff estimates based on IMF balance of payments data, and World Bank and OECD GDP estimates: Personal remittances, received (% of GDP). The World Bank; 2018. https://data.worldbank.org/indicator/BX.TRF.PWKR.DT.GD.ZS?locations=TJ. Accessed 3 Aug 2016.

[42] Steinmann P, Baimatova M, Wyss K (2012). Patient referral patterns by family doctors and to selected specialists in Tajikistan. Int Health.

