# Supplementary material for: Out of pocket expenditures of patients with a chronic condition consulting a primary care provider in Tajikistan: a cross-sectional household survey
Source: BMC Health Serv Res. 2020 Jun 16;20:546. doi: 10.1186/s12913-020-05392-2 (PMC7298845; doi:10.1186/s12913-020-05392-2)
Supplement: Supplementary file 1 — Additional file 1. Questionnaire [16]. [file 12913_2020_5392_MOESM1_ESM.pdf]

No.	Question	Codes
**1. Basic data**		
1.1	Patient Identification Number (from sample list taken from RHCs and constructed by research team)	Enter number
1.2	Patient consultation date	Day**/month**
1.3	Interviewer Code	Interviewers each given code (list kept with research team)
1.4	Reason for consultation? *Acute or chronic (chronic = longer than 3 months)*	1 = Acute (go to No. 1.5)<br>2 = Chronic (go to No. 1.6)<br>3 = Pregnancy (go to No. 1.7)<br>4 = Injury / Poison (go to No. 1.7)<br>5 = Other, specify (go to No. 1.7)
1.5	Which acute disease?<br>*After answering go to 1.7*	1 = Respiratory<br>2 = Digestive (gastrointestinal)<br>3 = Cardiovascular<br>4 = Diarrhoea<br>6 = Genitourinary<br>8 = Skin disease<br>9 = Anaemia<br>11 = Mental disorders<br>12 = Others, specify
1.6	Which chronic disease?<br>*After answering go to 1.7*	1 = Respiratory<br>2 = Digestive (gastrointestinal)<br>3 = Cardiovascular<br>4 = Cancer<br>6 = Genitourinary<br>8 = Skin disease<br>9 = Diabetes<br>10 = Anaemia<br>11 = Mental disorders<br>12 = Others, specify
1.7	Rayon Code	1 = Dangara<br>2 = Varzob<br>3 = Tursunzade<br>4 = Shakhinav<br>5 = Vose<br>6 = Khamadoni<br>7 = Rudaki<br>8 = Faizabad
1.8	Rural Health Centre Code	use codes from HMIS
**2. Patient experience**		
2.1 Experience with facility use (questions only target **the last visit** to the health facility)		
2.1.1	Is the distance from your home to the health centre acceptable?	1= yes, it's not a long distance and it's easy for me to come here<br>2= the centre is not close, but it's ok for me to come here<br>3= no, the centre is far away and it's difficult for me to come here
2.1.2	How far away is the health centre from your home?	1= less than 15 minutes<br>2= 15-29 minutes<br>3= 30-59 minutes<br>4= 60 minutes or more
2.1.3	Did you go to the health facility alone?	1 = Yes<br>2 = No<br>3 = I don't remember
2.1.4	Was this your first visit to this facility or was it a follow-up visit?	1 = I have never before been to this health facility<br>2 = I have been to this facility before, but for another health issue<br>3 = I have been to this facility before for the same health issue, so this was a follow-up visit
2.1.5	Were you at the health center more than once in the last month?	1 = Once<br>2 = Twice<br>3 = Three times<br>4 = More than three times<br>5 = I have not been in the last month
2.1.6	At the health centre, were you provided with information/leaflets that allows you to understand common diseases in your region, their treatment and prevention?	1 = Yes<br>2 = No<br>3 = Can’t remember
2.1.7	Do you regularly receive the health messages from the	1= yes, I regularly receive

No.	Question	Codes
	community groups, existing in your village and then discuss them among communities?	2= sometimes I receive<br>3= no, I don't receive<br>4= I'm not aware of such activities
2.2 Patient satisfaction		
2.2.1	Did the doctor examine your body as you expected him/her to do?	1= yes, he/she examined me<br>2= he/she did not do all the exams I though he/she would do<br>3= he/she did more than I thought was necessary/ than I expected him to do<br>4= no, he/she did not examined me at all<br>5= I do not remember
2.2.2	During your last visit, were you given adequate information on your health condition?	1 = Yes, very clear information/ explanations were given<br>2 = He / She could have explained in a better way<br>3 = No, not at all<br>4 = I did not need information
2.2.3	Do you feel that the doctor knows your patient's history and acts accordingly?	1 = Yes<br>2 = He could do better<br>3 = No, I need to explain to him everything again<br>4 = It was a new doctor<br>5 = I don't remember
2.2.4	During your last visit, were you given adequate information on your treatment?	1 = Yes, very clear information/ explanations were given<br>2 = He / She could have explained in a better way.<br>3 = No, not at all<br>4 = I did not need information
2.2.5	Overall, how satisfied are you with the care you received by the Family Doctor during this past visit? *(Instruction: Present sheet with possible answers (read out-loud if patient wishes))*	1 = Very satisfied<br>2 = Satisfied<br>3 = Neither satisfied nor dissatisfied<br>4 = Dissatisfied<br>5 = Very Dissatisfied
**3. OOP expenditures**		
3.1	Did the doctor send you to a specialist (polyclinic/hospital/specialized centres, dispensary, etc.)? *(Not lab-tests)*	1 = Yes<br>2 = No<br>3 = Can't remember
3.2	Did you have to pay any formal fees?	1 = Yes (go to 3.2.1)<br>2 = No (go to 3.3)
3.2.1	How much did you have to pay?	Value in Tajik Somoni<br>-99 = Don't know
3.3	Did you give any money to the Family Doctor or to the Family Nurse? *(Informal payment)*	1 = Yes (go to 3.3.1)<br>2 = No (go to 3.4)
3.3.1	How much money did you give to the Family Doctor or to the Family Nurse?	Value in Tajik Somoni<br>-99 = Don't know
3.4	Did you give any non-monetary gifts to the Family Doctor or to the Family Nurse? *(Informal payment)*	1 = Yes (go to 3.4.1)<br>2 = No (go to 3.5)
3.4.1	What was the approximate value of the gift?	Value in Tajik Somoni<br>-99 = Don't know
3.5	What resources did you use to pay for these informal expenditures? Where did the money come from?	1= Savings<br>2= Borrowing in Tajikistan<br>3=Borrowing/Remittances from relatives abroad<br>4= Credit<br>5=Sell farm products (animals, produce)<br>6= Sell valuables
3.6	How much money did you spend traveling to consult your family doctor?	Tajik Somoni<br>-99 = Don't know
3.7	Did the Family Doctor prescribe medicines for you during the consultation?	1 = Yes (go to 4.1)<br>2 = No (go to 4.6)<br>3 = I don't remember (go to 4.6)
**4. Medicine prescription and purchase**		
4.1	How many drugs were prescribed in total?	1=1<br>2=2<br>3=3<br>4=4<br>5=5<br>6=6<br>7=7<br>8= >=8
4.2	How many drugs were bought?	1= 1

No.	Question	Codes
		2=2<br>3=3<br>4=4<br>5=5<br>6=6<br>7=7<br>8= >=8<br>9 = I did not buy anything (go to 4.4)
4.2.1	Among the drugs bought, are there any transfusion (intravenous injection)?	1 = Yes<br>2 = No<br>3 = Don’t know
4.2.2	Among the drugs bought, are there any non-intravenous injection?	1 = Yes<br>2 = No<br>3 = Don’t know
4.2.3	Among the drugs bought, are there any antibiotics?	1 = Yes<br>2 = No<br>3 = Don’t know
4.2.4	Among the drugs bought, are there any vitamins?	1 = Yes<br>2 = No<br>3 = Don’t know
4.2.5	Considering the first drug, where did you buy it?	1= pharmacy<br>2= market<br>3= Family Doctor<br>4= hospital<br>5= other, specify
4.2.6	Considering the second drug, where did you buy it?	1= pharmacy<br>2= market<br>3= Family Doctor<br>4= hospital<br>5= other, specify
4.2.7	Considering the third drug, where did you buy it?	1= pharmacy<br>2= market<br>3= Family Doctor<br>4= hospital<br>5= other, specify
4.2.8	Considering the fourth drug, where did you buy it?	1= pharmacy<br>2= market<br>3= Family Doctor<br>4= hospital<br>5= other, specify
4.2.9	Considering the fifth drug, where did you buy it?	1= pharmacy<br>2= market<br>3= Family Doctor<br>4= hospital<br>5= other, specify
4.2.10	Considering the sixth drug, where did you buy it?	1= pharmacy<br>2= market<br>3= Family Doctor<br>4= hospital<br>5= other, specify
4.2.11	Considering the seventh drug, where did you buy it?	1= pharmacy<br>2= market<br>3= Family Doctor<br>4= hospital<br>5= other, specify
4.2.12	Considering the eighth drug, where did you buy it?	1= pharmacy<br>2= market<br>3= Family Doctor<br>4= hospital<br>5= other, specify
4.3	In total, how much money did you spend on these medicines?	Tajik Somoni<br>-99 = Don’t know
4.4	How much money did you spend traveling to obtain these medicines?	Tajik Somoni<br>-99 = Don’t know
4.5	What was the main reason why you did not obtain (all) the prescription medicines? *(if number of medicines bought<number of medicines prescribed)*	1 = No pharmacy near by<br>2 = No money<br>3 = Pharmacy did not have medicine in stock<br>4 = Did not feel I needed this medicine

No.	Question	Codes
		5 = other, specify
4.6	Did you use any other non-prescribed medicine?	1= Yes, from pharmacy<br>2= Yes, available in my house<br>3= Yes, other, specify<br>4= No
4.7	Did you use any supplementary treatment?	1= Yes, specify<br>2= No
**5 Demographics / asset index**		
5.1 General information		
5.1.1	Are you male or female?	1 = Male<br>2 = Female
5.1.2	What is your age?	In completed years
5.1.3	What is your main source of income household?	1= Private business<br>2= Salary<br>3= Pension or social aid<br>4= Remittances<br>5= Farming & livestock<br>6= Other
5.1.4	How many years of education have you undertaken?	1= Elementary (Grades 1-4)<br>2= Secondary incomplete (grades 5-8 (9))<br>3= Secondary complate (Grades 9-10 (11))<br>4= Specialized secondary\ Technical<br>5= Incomplete higher<br>6= Higher<br>7= Master<br>9= Graduate / Doctorate<br>98= Other, specify
5.1.5	In the past 12 months how many times have you visited a doctor for a consultation on a health related issue?	Number of visits
5.1.6	How would you rate your current health status?	1 = Very good (I rarely need to go to the doctor)<br>2 = OK (Sometimes I need to go and talk to the doctor)<br>3 = Poor (I very often/constantly need to see the doctor)<br>4 = Very poor (I can do almost nothing without being helped by a health professional)
5.1.7	How many people regularly live in the same house with you?	Number of household members
5.1.8	Do other people in the household have any chronic diseases?	Number of people with chronic conditions
5.1.9	Number of rooms in the house	Number of rooms
5.1.10	Main source of drinking water	1 = River, ditch or aryk<br>2 = Well or spring<br>3 = Public tap<br>4 = Tap at home<br>5 = Delivered from other place<br>6 = Mineral water<br>7 = Rain water<br>8 = Other
5.1.11	What type of toilet facility is being used by your household	1 = Hole<br>2 = Flushing toilet<br>3 = Public toilet<br>4 = No toilet
5.1.12	Do you have electricity at your house?	1 = Yes<br>2 = No
5.1.13	What material is the floor of the house	1 = Wooden<br>2 = Concrete with cover<br>3 = Loam (just earth)<br>4 = Other, specify
5.1.14	What material are the walls of the house	1 = Clay (loyi)<br>2 = Baked brick<br>3 = Cement (beton, stone)<br>4 = Other, specify
5.1.15	What is the occupation of the head of the household?	1 = farmer<br>2 = craftsmen (electrician, mason,…), “farosh”<br>3 = driver<br>4 = self-employed business<br>6 = teacher, administrative / professional, governmental employee<br>7 = unemployed

No.	Question	Codes
		8 = pensioner<br>9 = Working abroad<br>10 = other, specify
5.1.16	Where do you get your food from (mostly)?	1 = Market<br>2 = Home-grown<br>3 = Gifts/remittances (from aborad)
5.1.17	On average how many times in one month do you typically consume meat (during the time when you are healthy)?	1 = Zero<br>2 = Once per month<br>3 = 2-5 times per month<br>4 = 6-10 times per month<br>5 = Over ten times per month<br>6 = Every day<br>7 = Vegetarian
5.2 Does your household own ..... ? And if so, how many?		
5.2.1	bicycle?	1 = Yes (go to 5.2.1.1)<br>2 = No (go to 5.2.2)
5.2.1.1	If previous answer yes: How many?	Enter number of items
5.2.2	washing mashine?	1 = Yes (go to 5.2.2.1)<br>2 = No (go to 5.2.3)
5.2.2.1	If previous answer yes: How many?	Enter number of items
5.2.3	motorbike and/or scooter?	1 = Yes (go to 5.2.3.1)<br>2 = No (go to 5.2.4)
5.2.3.1	If previous answer yes: How many?	Enter number of items
5.2.4	car?	1 = Yes (go to 5.2.4.1)<br>2 = No (go to 5.2.5)
5.2.4.1	If previous answer yes: How many?	Enter number of items
5.2.5	truck?	1 = Yes (go to 5.2.5.1)<br>2 = No (go to 5.2.6)
5.2.5.1	If previous answer yes: How many?	Enter number of items
5.2.6	a refrigerator?	1 = Yes (go to 5.2.6.1)<br>2 = No (go to 5.2.7)
5.2.6.1	If previous answer yes: How many?	Enter number of items
5.2.7	an indoor heater (burzhuika)?	1 = Yes (go to 5.2.7.1)<br>2 = No (go to 5.2.8)
5.2.7.1	If previous answer yes: How many?	Enter number of items
5.2.8	a television?	1 = Yes (go to 5.2.8.1)<br>2 = No (go to 5.2.9)
5.2.8.1	If previous answer yes: How many?	Enter number of items
5.2.9	a DVD player?	1 = Yes (go to 5.2.9.1)<br>2 = No (go to 5.2.10)
5.2.9.1	If previous answer yes: How many?	Enter number of items
5.2.10	a satellite antenna/ dish?	1 = Yes (go to 5.2.10.1)<br>2 = No (go to 5.2.11)
5.2.10.	If previous answer yes: How many?	Enter number of items
5.2.11	a computer?	1 = Yes (go to 5.2.11.1)<br>2 = No (go to 5.2.12)
5.2.11.	If previous answer yes: How many?	Enter number of items
5.2.12	a phone (land and/or mobile phone)?	1 = Yes (go to 5.2.12.1)<br>2 = No (go to 5.2.13)
5.2.12.	If previous answer yes: How many?	Enter number of items
5.2.13	a watch?	1 = Yes (go to 5.2.13.1)<br>2 = No (go to 5.2.14)
5.2.13.	If previous answer yes: How many?	Enter number of items
5.2.14	an electric oven?	1 = Yes (go to 5.2.14.1)<br>2 = No
5.2.14.	If previous answer yes: How many?	Enter number of items
